# Area of Residence and Socioeconomic Factors Reduce Access to Biologics for Rheumatoid Arthritis Patients in Romania

**DOI:** 10.1155/2018/7458361

**Published:** 2018-05-08

**Authors:** Cătălin Codreanu, Claudiu C. Popescu, Corina Mogoşan

**Affiliations:** ^1^“Dr. Ion Stoia” Clinical Center for Rheumatic Diseases, 5th Thomas Masaryk Street, District 2, 020983 Bucharest, Romania; ^2^“Carol Davila” University of Medicine and Pharmacy, 37th Dionisie Lupu Street, District 1, 030167 Bucharest, Romania

## Abstract

**Introduction:**

The study aimed to evaluate the influence of socioeconomic factors on rheumatoid arthritis (RA) patients' access to biologics in Romania.

**Method:**

Cross-sectional data were collected in January 2014 from the Romanian Registry of Rheumatic Diseases (RRRD) comprising all RA patients on biologics from 42 Romanian counties. “Territorial” access to biologics was defined by patients receiving biologics in their home county. A county was “equitable” if <25% of RA patients received biologics outside it.

**Results:**

The RRRD included 4507 RA patients aged 56.7 ± 12.1 years, with a disease duration of 12.1 ± 8.3 years. Urban dwellers (67.8%) had a significantly higher prevalence of territorial biologic access than rural dwellers (83.1% compared to 74.1%; *p* < 0.001). Gross domestic product (GDP) in 1000 €/capita/county (odds ratio (OR) = 1.224) and number of physicians/1000 inhabitants/county (OR = 2.198) predict territorial access to biologics and also predict the number of territorially treated RA patients. Inequitable counties exhibited significantly lower socioeconomic indicators than equitable counties.

**Conclusion:**

In Romania, RA patients' access to biologics varies significantly between counties. Urban dwellers and patients living in counties/regions with high living standards are more likely to receive biologics locally than those living in more deprived areas.

## 1. Introduction

Rheumatoid arthritis (RA) is a systemic inflammatory autoimmune disorder affecting approximately 1% of the general population [1]. Recent epidemiological data show an increase in disease incidence in women in the past ten years [2]. RA can lead to functional disability, low quality of life, increased morbidity, and mortality [3].

Since RA usually affects professionally active people, it has important economic consequences, adding costs to patients and their families and to society through extensive use of health resources and loss of work productivity [4, 5]. The main goal of RA treatment is to induce and maintain remission whenever possible, or at least low disease activity [6]. Biological disease modifying antirheumatic drugs (bDMARDs) have revolutionized the therapy of RA and their efficacy is largely documented in clinical trials worldwide [7–9]. However, the high cost of bDMARDs is limiting their use, particularly in developing countries (since 2000, the Romanian public healthcare system reimburses adalimumab, etanercept, infliximab original and biosimilar, and rituximab, and since 2015 it also reimburses abatacept, certolizumab, golimumab, and tocilizumab).

In order to be eligible for bDMARDs in Romania, RA patients must be nonresponders to two different conventional synthetic DMARDs (csDMARDs) just like in other European countries, but they must fulfill other severity criteria which are stricter [10]. While some European countries do not reimburse bDMARDs yet, others offer free prescription, regardless of disease duration and previous therapies [10]. In 2011, among Central and Eastern European countries, Romania had the fifth highest prevalence of bDMARDs treatment among RA patients (2.2%) [11]. This variation of bDMARD uptake in Europe is coupled with observational evidence that socioeconomic status, area of residence, and income influence disease activity, disease outcome, and treatment access [12–15], which shows that significant inequities exist among different European countries with respect to RA management. Therefore, it is reasonable to hypothesize that these same limiting factors also create inequities at a national level especially because the regions of Romania differ in terms of socioeconomic and development characteristics. The Romanian territory is divided into 4 macroregions, 2 regions, and 42 counties: macroregion 1 consists of the Northwest (6 counties) and Center (6 counties) regions; macroregion 2 consists of Northeast (6 counties) and Southeast (6 counties) regions; macroregion 3 consists of South (6 counties) and Capital (2 counties) regions; macroregion 4 consists of Southwest (5 counties) and West (4 counties) regions. Historically and economically, macroregions 1 and 3 are the most developed areas of Romania, while macroregions 2 and 4 are significantly below the European Union's mean. This uneven distribution could hypothetically generate similar differences regarding access of RA patients to expensive medication (biologics). In this context, the study aims to evaluate the influence of socioeconomic factors on RA patients' access to biological therapy in Romania.

## 2. Materials and Methods

### 2.1. Romanian Eligibility Criteria for bDMARD Therapy in RA

In order to be considered for bDMARDs therapy, Romanian RA patients must fulfill four criteria: (a) the diagnosis of RA to be be made by a rheumatologist and it should fulfill the 2010 RA classification criteria [16]; (b) either high disease activity (HDA) irrespective of disease duration or early RA (under 2 years) with moderate disease activity (MDA) and with at least five poor prognosis factors, both with at least 5 swollen and/or tender joints and at least two of the following three criteria: morning stiffness above 60 minutes, erythrocyte sedimentation rate (ESR) above 28 mm/h, and C reactive protein (CRP) more than 3 times the upper limit of normal (ULN); (c) lack of response to at least two csDMARDs used for 12 weeks each; (d) no known contraindications for bDMARDs.

RA activity is assessed using the composite 28-joint count disease activity score (DAS28), which is based on the number of tender joints, number of swollen joints, ESR or CRP, and the visual analog scale for patient-reported general health [17]: patients with HDA have a DAS28 > 5.1, while patients with MDA have a DAS28 > 3.2.

The mentioned poor prognosis factors include age under 45 years; rheumatoid factor and/or anticitrullinated protein antibodies 10 times the ULN; ESR > 50 mm/h or CRP > 5 times the ULN; erosions on X-rays, ultrasound or magnetic resonance imaging; Health Assessment Questionnaire score above 1.5; extra-articular manifestations.

### 2.2. The Romanian Registry of Rheumatic Diseases (RRRD)

As soon as a patient fulfills the above criteria for bDMARDs use, the attending rheumatologist uploads the data into the RRRD, which is a national electronic database comprising all RA patients treated with biologics in Romania. Prior to treatment and inclusion in the RRRD, all patients signed an informed consent form for both bDMARD therapy and scientific use of their data.

For the purpose of this study, the variables were collected in January 2014 using a cross-sectional study design: demographics (age, sex, and area of residence) and RA-specific variables (disease duration, bDMARDs). The geographical distribution of patients (Romania has 42 administrative divisions: 41 counties and the capital) was reconfigured from a treatment perspective: if the patient received the bDMARD in home county, the case was considered “territorial” access; if the patient received the bDMARD outside home county, the case was considered “extraterritorial” access. Similarly, if a county had less than 25% of its RA patients treated outside, the county was considered to have “equitable” access; if more than 25% of its RA patients were treated outside, the county was considered to have “inequitable” access. The local and RRRD ethics committees approved the study protocol.

### 2.3. Socioeconomic Indicators

Socioeconomic indicators were collected from the National Institute of Statistics Yearbook (EUROSTAT [18]) and included the population distribution by county, indicators of living standards: gross domestic product (GDP) per capita per county in local currency (Romanian Leu) and Euro, physicians' distribution nationwide and by county, and the rheumatologists' distribution per counties.

### 2.4. Statistical Analysis

The normal distribution of the data was assessed using descriptive statistics, normality and stem-and-leaf plots, and the Lilliefors corrected Kolmogorov-Smirnov test. The age of the patients and their disease duration were distributed normally and therefore they were reported as “mean ± standard deviation.”

On a patient-based analysis (4507 patients), the association between residence (rural or urban) and bDMARD access (territorial and extraterritorial) was assessed using a *χ*^2^ test with a Cramer's *V* statistic for effect size. The differences between continuous variables (age, GDP, number of physicians, and number of rheumatologists) between patients with territorial or extraterritorial biologic access were assessed using *t*-tests. Effect size for these *t*-tests was calculated by Cohen's *d* statistic, approximated by running the *t*-tests using the standardized values (*Z* scores) of the independent variables and observing the mean difference output of these *t*-tests. A binary logistic regression model was computed in order to predict the likelihood that RA patients will have territorial bDMARD access using the following predictors: age (years), area of residence (coded “0” for rural and “1” for urban), GDP (expressed in 1000 €/capita/county) and number of physicians/1000 inhabitants/county. The results were reported in terms of odds ratios (OR) with 95% confidence intervals (CI).

On a county-based analysis (42 counties), GDP/capita, number of physicians/1000 inhabitants, and number of rheumatologists/county exhibited a nonnormal distribution; therefore the differences of these scale variables according to type of county (equitable and inequitable) were studied using Mann–Whitney tests (effect size for these tests was evaluated by estimating Glass rank biserial correlations using bivariate Spearman's correlations between the nominal and scale variables). For the same reason, the correlations between these scale variables among themselves and with the number of territorially and extraterritorially treated RA patients per county were computed using Spearman's rho coefficients. Two hierarchical multiple linear regression models were constructed in order to predict the number of RA patients with territorial access to biologics. Both models used GDP/capita as predictor in the first step. For the second step, in the first model the number of physicians/1000 inhabitants was added as predictor, while in the second model the number of rheumatologists/county was added as predictor. These predictors were previously normalized by extracting square roots (number of rheumatologists/county) or by calculation of their natural logarithms (number of physicians/1000 inhabitants). Since the number of physicians and the number of rheumatologists are not independent variables (the number of physicians includes the number of rheumatologists), they were not included together in the same regression model.

The statistical tests were considered significant if *p* < 0.05. All the statistical analysis was done using IBM SPSS Statistics version 22.0 for Windows (Armonk, NY, IBM Corp.).

## 3. Results

### 3.1. Patient-Based Analysis

**Figure 1:**
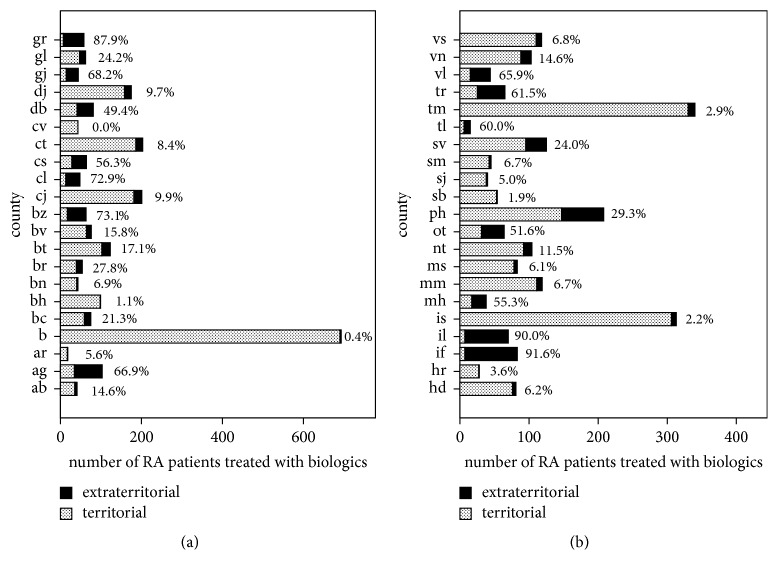
The distribution of RA patients treated with biologics according to county and treatment access (territorial and extraterritorial). The percentages represent the fraction of RA patients who benefited from extraterritorial access to biologics: for example, in the capital (“b”, (a)), there were 693 RA patients on biologics, but only 3 (0.4%) were treated extraterritorially, while in other counties more than 90% were treated extraterritorially. Counties are designated by their Romanian abbreviation.

**Figure 2:**
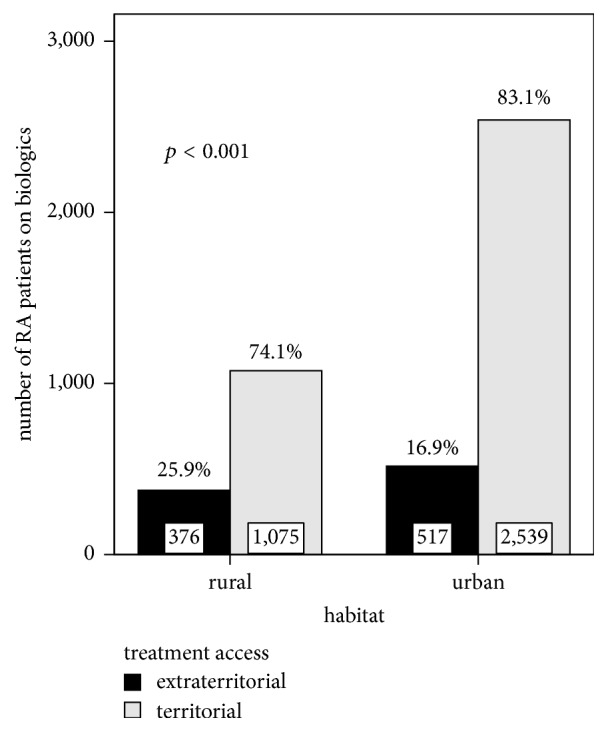
Access to biologics of RA patients according to habitat: 83.1% of urban dwellers had territorial access to biologics, compared to only 74.1% of rural dwellers (*p* < 0.001; *χ*^2^ test; effect size Cramer's *V* = 0.205, *p* < 0.001).

Until January 2014, the RRRD included 4507 patients with RA: 4267 (94.7%) treated with biologics and 240 (5.3%) with approved biological therapy, but treatment not yet started. The patients had a mean age of 56.7 ± 12.1 years and a mean RA duration of 12.1 ± 8.3 years. The majority of patients were women (3842 patients—85.2%) and the majority of patients lived in urban areas (3056 patients—67.8%). In the sample, 80.2% (3614) of RA patients benefited from territorial bDMARD access, while 19.8% (893) had extraterritorial bDMARD access (Figure 1, Table 1). Urban dwellers had a significantly higher prevalence of territorial bDMARD access than rural dwellers (Figure 2).

**Figure 3:**
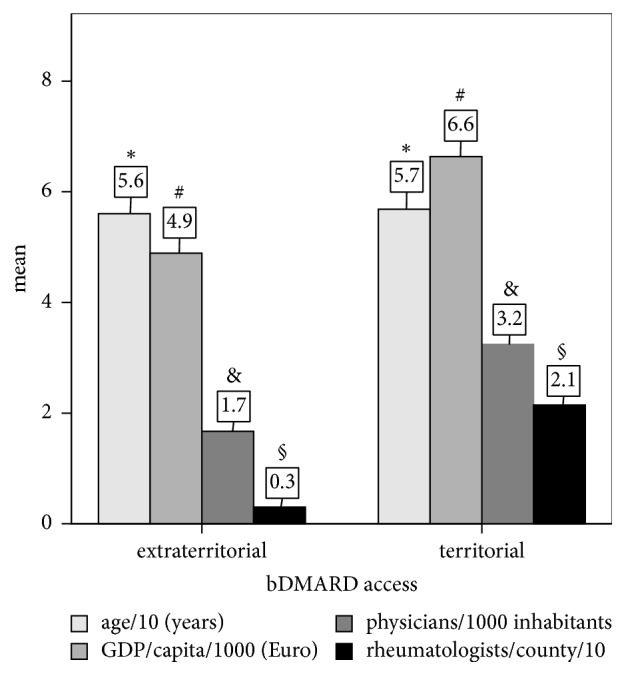
Differences between RA patients with territorial or extraterritorial access to biologics (bDMARD) regarding age and county socioeconomic indicators (gross domestic product – GDP/capita; number of physicians/1000 inhabitants and number of rheumatologists). The *p* values represent the significance of *t*-tests. Effect sizes are evaluated by Cohen's *d* statistics: −0.72 for rheumatologists/county, −0.89 for physicians/county; −0.51 for GDP/capita; −0.07 for age. The variables have been scaled to appropriate coillustration size by decimal division. ^#, &, §^*p* < 0.001; ^*∗*^*p* = 0.067.

Compared to RA patients with territorial bDMARD access, those with extraterritorial bDMARD access had equivalent mean ages but came from counties with significantly lower socioeconomic indicators (Figure 3).

The logistic regression model (*χ*^2^(5) = 1018.9; *p* < 0.001) explained 32.1% of bDMARD accessibility (Nagelkerke *R*^2^ = 0.321) and it correctly classified 81.7% of patients. Accounting for all other predictors included/present in the model, GDP expressed in 1000 €/capita/county (OR = 1.224; 95% CI: 1.186–1.263) and number of physicians/1000 inhabitants per county (OR = 2.198; 95% CI: 1.845–2.618) significantly increased the likelihood that patients will have (equitable) territorial bDMARD access.

### 3.2. County-Based Analysis

**Figure 4:**
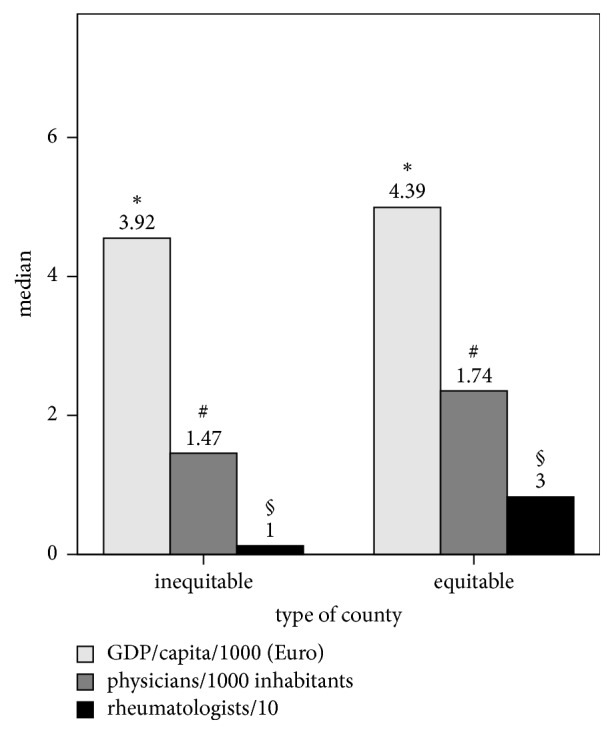
Differences between equitable and inequitable counties regarding access to biologics in terms of socioeconomic indicators (gross domestic product – GDP/capita; number of physicians/1000 inhabitants and number of rheumatologists). The *p* values represent the significance of Mann–Whitney tests. Effect size was evaluated using Glass rank biserial correlation: 0.34 for GDP/capita; 0.38 for physicians; 0.63 for rheumatologists. The variables have been scaled to appropriate coillustration size by decimal division. ^*∗*^*p* = 0.338, ^#^*p* = 0.016, and ^§^*p* < 0.001.

The majority of RA patients who are treated with bDMARDs outside their home environments chose the capital (Bucharest): 53.7% (805/1498) of patients treated in Bucharest come from a different county. Compared to equitable counties regarding bDMARD treatment, the inequitable counties exhibited significantly lower socioeconomic indicators (Figure 4).

The number of rheumatologists/county, the number of physicians/1000 inhabitants/county, and GDP/capita were significantly and positively correlated with the number of territorially treated RA patients/county (rho = 0.843, *p* < 0.001; rho = 0.448, *p* = 0.003; rho = 0.337, *p* = 0.034, resp.), and they were significantly and negatively correlated with the number of extraterritorially treated RA patients/county (rho = −0.340, *p* = 0.027; rho = −0.410, *p* = 0.007; rho = −0.337, *p* = 0.034 resp.).

The distribution of socioeconomic indicators among counties was uneven in terms of number of rheumatologists/county (mean = 5.6; median = 2.0; skewness = 4.84; kurtosis = 26.2; minimum = 0; maximum = 75), the number of physicians/1000 inhabitants/county (mean = 2.01; median = 1.52; skewness = 2.13; kurtosis = 4.48; minimum = 1; maximum = 6.02), and GDP/capita (mean = 4824 €; median = 4299 €; skewness = 2.48; kurtosis = 8.25; minimum = 2535 €; maximum = 13575 €).

Two hierarchical regression analysis models were computed to predict the number of territorially treated RA patients using GDP (1000 €/capita/county) in the first step and then adding either the number of physicians/1000 inhabitants/county or the number of rheumatologists/county as predictors (Table 2). GDP on its own significantly explained 43.4% of the variance of territorially treated RA patients. Adding either the number of physicians/1000 inhabitants/county or the number of rheumatologists/county produced significant models in which these variables predicted an additional 22.3% and 46.8%, respectively, in the variance of territorially treated RA patients. In both models GDP became an insignificant predictor when both independent variables (GDP and number of physicians or GDP and number of rheumatologists) were entered into the regression model, an observation explained by the degree of correlations of GDP with the number of physicians/1000 inhabitants/county (rho = 0.731; *p* < 0.001) and with the number of rheumatologists/county (rho = 0.438; *p* = 0.004).

## 4. Discussion

The aim of the study was to assess the impact of socioeconomic factors on Romanian RA patients' accessibility to bDMARDs. In this sense, we found that rural habitat and poor county socioeconomic indicators (GDP/capita, number of physicians/1000 inhabitants, and number of rheumatologists/county) are associated with lower access to bDMARDs. In order to discuss the relevance of these results, we must first review the characteristics of the sample. Compared to patients from other European RA registries [19], RA patients from the RRRD have an equivalent mean age, the same form of established disease according to disease duration, but a higher prevalence of female patients. In the absence of conclusive epidemiological studies of RA in Romania, this observation may be explained by a number of competing reasons: underdiagnosis of men; higher disease severity among women requiring biological therapy; a hypothesized genetic population trait. Detailed prevalence studies are needed to assess these possibly non-mutually exclusive hypotheses.

Observational studies have reported that financial factors such as macroeconomic conditions, income, and national health expenditure are major influencing factors of bDMARD accessibility in European countries [11, 15, 20–23]. Even though these studies made observations by comparing different countries, it is reasonable to expect that the same financial factors influence bDMARD accessibility of RA patients within different regions of the same country, given the existence of macroeconomic heterogeneity between these regions. As EUROSTAT data show, the 41 counties and the capital of Romania display an important amount of GDP/capita heterogeneity. Given these significant differences of GDP/capita among Romanian counties, we indeed showed that low GDP/capita predicts low bDMARD access. Furthermore, even though national guidelines attempt to create equal opportunities in terms of access to biologic therapy for all eligible RA patients, in practice many patients need to travel to another county in order to benefit from bDMARDs. Finally, following its highest GDP/capita among counties, we observed a strong centralizing effect of the capital on the number of RA patients treated with bDMARDs, a tendency noted by other authors in the literature [24]. Since every county has its own Health Insurance House which reimburses treatment cost, a revision of the distribution of funds would possibly increase accessibility to bDMARDs outside the capital.

The observed predictive power of the number of physicians/rheumatologists for bDMARD access of RA patients is intrinsically linked to the characteristics of the disease and the structure of the health system. In this sense, the number of physicians has been long used as an indicator of socioeconomic development. Nationwide, for a population of roughly twenty million inhabitants, there were 235 active rheumatologists. Out of the 42 administrative divisions of Romania, there were 7 counties without a rheumatologist, while in the capital there were 75 active rheumatologists. The distribution of rheumatologists among the 41 counties and the capital was uneven and it was correlated with territorial economic level, as measured by the GDP/county (rho = 0.731; *p* < 0.001). While other more economically developed countries are also facing a shortage of rheumatologists [25, 26], the proportions are very different. The lack of specialized healthcare professionals in rheumatology leads to known barriers to bDMARD therapy in RA, such as long waiting time for medical visits and travel difficulties related to long distances to rheumatology clinical settings [27, 28]. A study investigating patient-reported barriers to access bDMARD treatment in Romanian RA patients would assess the full extent of the issue.

There are some limitations of this study which could influence interpretation of the results. There were no data regarding educational status (a known influencing factor of bDMARD access) [15] and the actual extent of disease activity above the protocol cutoff (DAS > 5.1). A geographical confounder, which we were unable to control, can be described, namely, the travel distances to clinics between different counties: these must have been patients who lived very close to county borders and therefore their extraterritorial bDMARD access could have been a matter of convenience. Additional variables (such as socioeconomic status of each patient and the number of patients who did not receive biologics because of travelling limitations) were not collected. All the analyzed variables came from different contributors to the RRRD (the attending rheumatologists) and their quality relies on the assumption of correct data input into the national electronic system.

## 5. Conclusions

In Romania, accessibility of RA patients to biological therapy varies significantly between different counties. Areas with low socioeconomic level do not offer equal and fair therapeutic opportunities for RA patients compared to other national areas: patients with RA living in urban areas and counties/regions with high living standards are more likely to receive biological agents locally than those living in more deprived areas. Studies investigating patient-reported barriers to biologic therapy are needed in the Romanian population.

## Figures and Tables

**Table 1:** Characteristics of Romanian divisions: macroregions (4), regions (8), and counties (42).

Division	Inhabitants	GDP	Physicians	Rheumatologists	RA biologics
**Macroregion 1**	**5468525**	**7123.2**	**2.45**	**50**	**872**
*Northwest*	*2834186*	*7049.7*	*2.72*	*32*	*547*
Bihor	619102	5095.0	2.93	6	99
Bistriţa-Năsăud	329188	4503.3	1.33	2	43
Cluj	721955	6977.6	5.08	16	201
Maramureş	525765	3809.4	1.56	3	119
Satu Mare	390639	4014.1	1.52	2	45
Sălaj	247537	4304.7	1.51	3	40
*Center*	*2634339*	*7406.4*	*2.29*	*18*	*325*
Alba	380976	5320.4	1.77	1	41
Braşov	630807	6521.9	2.16	5	76
Covasna	228732	4288.3	1.70	1	43
Harghita	333674	4361.5	1.38	1	28
Mureş	595948	4428.3	3.50	8	83
Sibiu	464202	6358.1	2.54	2	54

**Macroregion 2**	**6794269**	**6235.8**	**1.68**	**64**	**1358**
*Northeast*	*3922407*	*4887.6*	*1.72*	*45*	*858*
Bacău	746566	3899.3	1.43	3	75
Botoşani	455973	2922.3	1.33	2	123
Iaşi	919049	4227.4	3.25	30	313
Neamţ	577359	3118.8	1.40	4	104
Suceava	743645	3375.7	1.34	4	125
Vaslui	479815	2535.4	1.29	2	118
*South-East*	*2871862*	*7161.8*	*1.66*	*19*	*500*
Brăila	356196	4519.9	1.47	2	54
Buzău	478811	3582.4	1.19	1	63
Constanţa	769768	6414.3	2.62	10	203
Galaţi	631669	3766.2	1.38	2	62
Tulcea	244249	3755.8	1.47	1	15
Vrancea	391169	3262.1	1.31	3	103

**Macroregion 3**	**5757871**	**10892.3**	**3.22**	**89**	**1469**
*South*	*3260976*	*6724.4*	*1.38*	*13*	*633*
Argeş	646333	6487.6	2.11	3	103
Călăraşi	317293	3223.0	1.00	0	48
Dâmboviţa	528426	4090.3	1.23	2	81
Giurgiu	276781	3291.2	1.06	0	58
Ialomiţa	293658	3747.3	1.01	0	70
Prahova	809052	5828.3	1.45	7	208
*Capital*	*2496895*	*18653.8*	*4.74*	*76*	*776*
Ilfov	390751	9798.3	1.51	1	83
Bucureşti	2106144	13574.6	6.02	75	693

**Macroregion 4**	**4221053**	**6706.5**	**2.82**	**32**	**868**
*Southwest*	*2206321*	*5683.5*	*2.10*	*9*	*365*
Dolj	700117	4486.8	3.02	7	175
Gorj	366261	5498.3	1.75	0	44
Mehedinţi	286678	3527.0	1.70	0	38
Olt	450094	3090.2	1.49	0	64
Vâlcea	403171	4292.8	1.75	2	44
*West*	*2014732*	*8045.6*	*3.13*	*23*	*503*
Arad	473946	5599.7	2.44	3	18
Caraş-Severin	328047	4703.2	1.79	0	64
Hunedoara	469853	4742.9	2.36	5	81
Timiş	742886	7938.1	4.99	15	340

*Notes*. Data from 2013; county names are in Romanian; for each county: GDP €/capita, number of physicians/1000 inhabitants, absolute number of rheumatologists, and absolute number of RA patients treated with biologics. GDP: gross domestic product; RA: rheumatoid arthritis.

**Table 2:** Hierarchical regression models predicting the number of territorially treated RA patients.

	GDP	GDP + physicians	GDP + rheumatologists
*R* ^2^	0.434	0.656	0.901
*F*	30.6	37.3	177.9
*p*_*R*_^2^	<0.001	<0.001	<0.001
*R* ^2^ change	-	0.223	0.468
*p*_*R*_^2^ change	-	<0.001	<0.001
*B*	93.1	72.1	−3.7
*p*_*B*_	<0.001	0.203	0.718

*Notes*. GDP was expressed per 1000 €/capita/county; the number of physicians was expressed per 1000 inhabitants/county; the number of rheumatologists was expressed per county; there are 2 models: model 1 (GDP at the first step, GDP and physicians at the second step); model 2 (GDP at the first step, GDP and rheumatologists at the second step). GDP: gross domestic product; RA: rheumatoid arthritis.
